# Development and convergent validity of new self-administered questionnaires of active transportation in three African countries: Kenya, Mozambique and Nigeria

**DOI:** 10.1186/s12889-018-5954-z

**Published:** 2018-08-16

**Authors:** Vincent O. Onywera, Richard Larouche, Adewale L. Oyeyemi, Antonio Prista, Kingsley K. Akinroye, Sylvester Heyker, George E. Owino, Mark S. Tremblay

**Affiliations:** 1 Department of Recreation Management and Exercise Science, Kenyatta University, P.O BOX 43844-00100, Nairobi, Kenya; 2 Faculty of Health Sciences, University of Lethbridge, Lethbridge, Canada; 3 Department of Physiotherapy, University of Maiduguri, Maiduguri, Nigeria; 4 Physical Activity and Health Research Group, Research Center on Physical Activity and Sports, Universidade Pedagogica, Maputo, Mozambique; 5 Nigerian Heart Foundation, Lagos, Nigeria; 6 Department of Leisure and Event Management, The Technical University of Kenya, Nairobi, Kenya; 7 Department of Sociology, Kenyatta University, Nairobi, Kenya; 8 Healthy Active Living and Obesity Research Group, Children’s Hospital of Eastern Ontario Research Institute, Ottawa, Canada

**Keywords:** Physical activity, Walking, Cycling, Psychometrics, Children, Africa

## Abstract

**Background:**

There is currently a rapid physical activity transition taking place in developing countries that includes a decrease in active transportation. Building on findings from an earlier systematic review, this paper describes the development and convergent validity of self-administered child and parent questionnaires assessing active transportation of children in three African countries: Kenya, Mozambique and Nigeria.

**Methods:**

A pilot study was conducted to examine the convergent validity of the developed questionnaires by comparing responses between children and their parents (*N* = 121; *n* = 43 for Mozambique, *n* = 24 for Kenya and *n* = 54 for Nigeria). After modification, the questionnaires were then administered to a larger convenient sample of both children and parents from Kenya (*n* = 1123), Mozambique (*n* = 1097) and Nigeria (*n* = 831) which defined the main study. The questionnaires assessed active transportation to/from 8 categories of destinations including school, friends’ and relatives’ home/houses, parks and playgrounds among others. Twenty items were used to assess child - and parent-perceived barriers to active transportation, and the parent questionnaire inquired about parent education and availability of cars, motorcycles, and bicycles. Spearman’s rho was used to compare children’s mode of travel in the pilot study while the prevalence-adjusted bias-adjusted kappa (PABAK) coefficient was used to compare convergent validity between children’s and parents responses on active transportation in the main study.

**Results:**

Findings of the main study show that convergent validity for active transportation to and from each destination in the combined sample ranged from 0.472 (from school) to 0.998 (to other places). Convergent validity for challenges/barriers to active transportation to school ranged from fair (0.30 - The route does not have good lighting) to substantial (0.77 - My child has a disability). It varied between countries from fair (*n* = 11-items) to moderate (*n* = 9-items) agreement in Kenya and from poor (*n* = 2-items) to fair (*n* = 16-items) agreement in Nigeria. Data from Mozambique was however missing and therefore could be included.

**Conclusions:**

The questionnaires provided valid information on the number of trips to/from various destinations and show acceptable and modest convergent validity for measuring barriers to active transport in a sample of children from three African countries. These questionnaires may be suitable for future research on active transport among school children in Sub-Saharan African countries.

**Electronic supplementary material:**

The online version of this article (10.1186/s12889-018-5954-z) contains supplementary material, which is available to authorized users.

## Background

The World Health Organization recommends that children and youth accumulate at least 60 min of moderate-to-vigorous physical activity (MVPA) daily [1]. However, a growing body of evidence suggests that the majority of children and youth worldwide do not meet this recommendation [2–6]. Collating data from 105 countries across the world, Hallal and colleagues [5] reported that only 20% of 13–15 year-olds were sufficiently active. Data from African countries who participated in the Health Behavior in School-aged Children survey indicate that only 8% to 35% of African youth engaged in MVPA for 60 min on at least 5 days per week [4]. Insufficient MVPA is associated with the development of cardiovascular disease risk factors in childhood and adolescence [7], and with a substantial proportion of the global burden of disease in adulthood [8].

In the African context, a large proportion of overall physical activity (PA) is thought to be accumulated through the use of non-motorized travel modes (i.e., active transportation; AT). Nevertheless, few studies have assessed AT among African children and youth, and the majority of these studies have focussed exclusively on the trip to/from school [9]. While they have been overlooked in the AT literature, other destinations, including parks, sport fields, markets, friends’ and relatives’ houses, may present important opportunities to engage in AT [10]. This is of particular interest because there is consistent evidence showing that active travelers are more active than motorized travelers [11]. Furthermore, AT may have other environmental co-benefits, such as a reduction in exhaust gas emissions which contribute to climate change and are involved in the pathophysiology of cardiovascular and respiratory diseases [12].

As a prerequisite to examining the contribution of AT to overall PA, it is important to develop valid and reliable measures. To date, there is evidence stemming mostly from high-income countries that self- or parent-reported measures of AT to/from school show high test-retest reliability and convergent validity between child and parent responses, but very few researchers have investigated the psychometric properties of measures of AT to/from other destinations [9]. In this paper, convergent validity refers to the degree to which the parent and child responses match in relation to measures of active transportation. Given the role and influence that parents/guardians have on children, it was important to assess the convergent validity between parents and children. To date, only Oyeyemi and colleagues [13] have reported the test-retest reliability of a measure of AT in an African sample, specifically Nigerian adolescents aged 12–18 years, and their results show modest reliability (ICC = 0.45) [14]. To our knowledge, no data on the psychometric properties of measures of AT among African children are available.

Therefore, the purpose of this paper was to report on the development and validation of new self-administered questionnaires (child and parent) to assess AT to/from school as well as other destinations among children in three African countries representing the Eastern, Western and Southern regions of Africa: Kenya, Nigeria and Mozambique.

## Methods

### Participants

Ethical approval was obtained from Kenyatta University Ethics Review Board, Nigerian Heart Foundation Ethics Committee, Pedagogical University Ethics Board and the Children’s Hospital of Eastern Ontario Research Ethics Board. This project involved multiple phases: 1) an environmental scan of the literature on the psychometric properties of AT measurement tools; 2) the development of a tool for the African context involving researchers from multiple African countries; 3) testing of the tool in a pilot study; 4) refinement of the tool based on expert consensus; and 5) testing of the tool in a larger (main) study. One hundred and twenty one children (Mean age = 11.2; 10–12 years) from urban (47.2%), suburban (26.4%) and rural (26.4%) areas of Kenya, Mozambique and Nigeria participated in the pilot study. Participants were purposively recruited and sampled according to urban, suburban and rural areas in Mozambique and Nigeria, but only from an urban area in Kenya. The main study involved 3051 participants living in urban, suburban and rural areas of Kenya (*n* = 1123), Mozambique (*n* = 1097) and Nigeria (*n* = 831).

### Procedure

#### Development of the questionnaires

The first step of the project was to conduct an environmental scan of tools to assess AT. This involved a systematic review of existing instruments and methods that have been used to assess AT in children and youth in different areas of the world [9]. The review was not restricted to the trip to/from school. This review revealed a dearth of information and research on active travel behaviours among African children and youth and highlighted the need to develop valid measures of non-school active travel for this African population. Accordingly, the research/experts team met in early 2013 in Nairobi, Kenya and in 2014 in Maputo, Mozambique to discuss the relevance to Africa of available AT instruments and develop a standardized data collection procedure. These consensus meetings also aimed to uphold the content validity of the instruments and ensure that potential destinations for AT in the African context are included in the questionnaires.

The research team was unable to identify any AT instrument, questionnaire or method that completely captured the African context and was suitable for assessing AT among African children and youth. Therefore, the team developed new child and parent questionnaires to assess AT in African children and youth, including whether mode of travel to school differed between seasons in the African countries. The AT questionnaires that were developed (Additional file 1) contain 10 broad questions for parents and six broad questions for children including questions on demographics, mode of travel to and from school during different school terms (seasons), perceived distance between home and school, and a question that includes 20 items assessing perceived barriers to AT that was adapted from Forman and colleagues. There are also questions on the number of trips to and from various destinations including school, friend’s house/home, relative’s houses/home, parks or playgrounds, shops or markets, or restaurants, sport venues (soccer field, swimming pool), faith places (such as church, mosque) and others. The questionnaires were translated from English into other official or local languages in the three countries where the project took place, for instance in Kenya (Kiswahili), Nigeria (Yoruba) and Mozambique (Portuguese) and back-translated to ensure content preservation of the questionnaire. The translation of questionnaires was done by professional translators while the back translation was done by English speakers who also have an understanding of the cultural context.

Following the translation process, a pilot study was conducted among a convenience sample of 121 children aged 10–12 years from three African countries (Kenya (*n* = 24), Nigeria (*n* = 54) and Mozambique (*n* = 43) to assess the convergent validity between child and parent reports and assess respondent comprehension. Convergent validity was operationalized as the degree of agreement between children and their parents. Presumably, high convergent validity would increase our confidence that children and parents are capable of responding to these questions accurately. The questionnaires were modified accordingly based on the results of the pilot study. Modifications to the questionnaires included elimination of questions on seasonality and change of response options for questions on perceived barriers from 4-point rating scale (from strongly disagree to strongly agree) to a yes/no format. The latter change was made because participants expressed difficulty in understanding terms such as “somewhat agree” during the pilot study. After modification, the questionnaires were administered to a larger convenience sample of both children and parents from Kenya (*n* = 1123), Mozambique (*n* = 1097) and Nigeria (*n* = 831) living in urban, peri-urban and rural areas to further assess the convergent validity between child and parent reports. Mozambique did not however collect information on barriers. The three countries were chosen because they represent Eastern, Western and Southern African regions hence increasing the generalizability of the findings to other countries in Sub-Saharan Africa.

### Statistical analyses

For the pilot study, Spearman’s rho was used to compare children’s main school travel mode across seasons. The convergent validity between children and parent’s responses for children’s primary school travel mode was assessed using Cohen’s kappa [15]. Quadratic weighted kappa [16] were used for the questions on perceived barriers to AT given the ordinal response options. The convergent validity of the number of trips to and from each destination, as reported by parents and children, was assessed with intra-class correlation coefficients (ICC), using the “two-way mixed” procedure for a single measure for absolute agreement [17] with a 95% confidence interval. The guidelines proposed by Landis and Koch [15] were used to qualify the kappa and ICC coefficients as slight (< 0.20), fair (0.21**–**0.40), moderate (0.41–0.60), substantial (0.61–0.80) or almost perfect (0.81–1.00). Similar analyses were performed for the main study, except for the dichotomized perceived barriers which were assessed with Cohen’s [16] kappa rather than weighted kappa. Because kappa coefficients are known to be affected by the distribution of the variable, the prevalence-adjusted bias-adjusted kappa (PABAK) coefficient was provided as a complement to Cohen’s kappa [18, 19]. Being continuous data, the interclass correlation coefficient was used to analyze data for the various country strata (urban, peri-urban and rural). The analyses were done on the whole sample (all countries combined) and separately for Kenya and Nigeria. Country specific analysis for Mozambique was not presented due to missing data because parent questionnaires were not administered. All analyses were conducted with SPSS version 23 (Armonk, NY, USA). Missing data were deleted listwise. Multiple imputation was not used to address cases of missing data because it was not suitable in the context of a validation study.

## Results

In the pilot study, very high correlations were observed for children’s main travel mode to and from school in different seasons, whether they were reported by parents (rho from 0.901 to 0.956; all *p* < 0.001) or by children (rho from 0.827 to 0.965; all *p* < 0.001). Substantial agreement was noted between children’s and parents’ report on the main school travel mode for each season (κ from 0.665 to 0.736; all *p* < 0.001). Furthermore, substantial convergent validity was noted for the number of reported trips to and from most destinations with the exception of sport venues for which agreement was only slight to moderate. Convergent validity was only slight to moderate for the perceived barriers to AT (weighted κ from 0.015 to 0.531). Nevertheless, it is worth noting that relatively higher values were observed for barriers such as long distance, convenience and traffic. Given the very high correlations between seasons (See Additional file 2), questions about seasonality were not included in the main study.

In the main study, moderate (0.472) to substantial (0.998) convergent validity was observed between child and parent reports for children’s number of trips to and from various destinations as can be seen in Table 1. The least valid and most valid destinations were ‘from school’ (0.472) and to ‘other places’ (0.998) respectively. It is worth noting that few participants reported trips to and from ‘other places’. It is likely that few participants travelled to ‘other places’ because our questionnaire covered various destinations comprehensively.

**Table 1:** Convergent validity for the number of active trips to and from each destination -parent vs. child report (main study, *n* = varies for each variable)

	N	ICC	95% CI
Active transportation to			
School (Parents) vs School (Child)	1985	0.546	0.514–0.576
Friends (Parents) vs Friends (Child)	1928	0.627	0.750–0.791
Relatives (Parents) vs Relatives (Child)	1922	0.665	0.639–0.689
Park (Parents) vs Park (Child)	1917	0.609	0.580–0.636
Shop (Parents) vs Shop (Child)	1944	0.582	0.552–0.611
Sport (Parents) vs Sport (Child)	1901	0.568	0.537–0.597
Faith (Parents) vs Faith (Child)	1954	0.706	0.683–0.728
Active transportation from			
School (Parents) vs School (Child)	1977	0.472	0.437–0.505
Friends (Parents) vs Friends (Child)	1921	0.597	0.568–0.625
Relatives (Parents) vs Relatives (Child)	1919	0.612	0.583–0.639
Park (Parents) vs Park (Child)	1912	0.615	0.587–0.643
Shop (Parents) vs Shop (Child)	1940	0.605	0.576–0.633
Sport (Parents) vs Sport (Child)	1896	0.568	0.511–0.575
Faith (Parents) vs Faith (Child)	1947	0.706	0.710–0.752

The convergent validity between child and parent reports on perceived barriers to AT ranged from fair agreement with lack of ‘suitable walking/running or biking paths’ (k = 0.36) to substantial agreement with ‘my child has disability’ (k = 0.77) as shown in Table 2. Further Table 3 shows the country specific (for Kenya and Nigeria) convergent validity between child and parents reports on perceived challenges/barriers to AT to and from school. There was large inter-country differences in convergent validity. It ranged between fair (*n* = 11-items) to moderate (*n* = 9-items) agreement in Kenya and from poor (*n* = 2-items) to fair (*n* = 16-items) agreement in Nigeria. Results also show a high convergent validity for the number of active trips to/from each destinations across the three environmental strata (urban, peri-urban and rural), with the exception of peri-urban areas in Kenya, as presented in Tables 4, 5, 6 and 7.

**Table 2:** Bennett’s kappa PABAK analysis of challenges/barriers of active transport to school (pooled sample of Kenya and Nigeria) -parent vs. child report

Item	n	Bennett’s Kappa	Strength of Agreement
There are too many hills along the way	2012	0.64	Substantial
There are no suitable walking/running or biking paths	2002	0.39	Fair
The route is boring (nothing interesting to see)	2025	0.43	Moderate
The route does not have good lighting	1997	0.30	Fair
There is too much traffic along the route	2005	0.37	Fair
There are dangerous crossings	1997	0.41	Moderate
My child gets too hot and sweaty	1993	0.37	Fair
No other children walk/run or bike to school	1983	0.48	Moderate
It’s not considered fashionable to walk/run or bike	1088	0.48	Moderate
My child has too many things to carry	1992	0.51	Moderate
It is easier for me to drive my child	1972	0.46	Moderate
It involves too much planning ahead	1964	0.40	Fair
It is unsafe because of crime (strangers, gangs, drugs)	1988	0.36	Fair
My child gets bullied, teased, harassed	1977	0.53	Moderate
There is nowhere to leave a bike safely	1993	0.36	Fair
There are stray dogs or other dangerous animals	1986	0.45	Moderate
It is too far	2000	0.43	Moderate
The route is difficult to walk/run or bike because of garbage, water or bad smells	1997	0.42	Moderate
The route is isolated	1988	0.42	Moderate
My child has a disability	1996	0.77	Substantial

**Table 3:** Bennet’s kappa analysis of challenges/barriers to active transport to school (Kenya and Nigeria analyzed separately) -parent vs. child report

Item	Kenya			Nigeria		
	N	Bennett’s Kappa	Strength of Agreement	N	Bennett’s Kappa	Strength of Agreement
There are too many hills along the way	1093	0.58	Moderate	919	0.44	Moderate
There are no suitable walking/running or biking paths	1093	0.32	Fair	910	0.35	Fair
The route is boring (nothing interesting to see)	1090	0.47	Moderate	911	0.26	Fair
The route does not have good lighting	1087	0.21	Fair	911	0.21	Fair
There is too much traffic along the route	1092	0.33	Fair	915	0.35	Fair
There are dangerous crossings	1085	0.42	Moderate	916	0.36	Fair
My child gets too hot and sweaty	1084	0.43	Moderate	909	0.31	Fair
No other children walk/run or bike to school	1087	0.31	Fair	897	0.21	Fair
It’s not considered fashionable to walk/run or bike	1087	0.47	Moderate	Nil	Nil	Nil
My child has too many things to carry	1086	0.59	Moderate	906	0.28	Fair
It is easier for me to drive my child	1079	0.39	Fair	893	0.23	Fair
It involves too much planning ahead	1083	0.33	Fair	891	0.21	Fair
It is unsafe because of crime (strangers, gangs, drugs)	1088	0.24	Fair	910	0.28	Fair
My child gets bullied, teased, harassed	1083	0.36	Fair	904	0.26	Fair
There is nowhere to leave a bike safely	1089	0.38	Fair	905	0.06	Slight
There are stray dogs or other dangerous animals	1083	0.48	Moderate	913	0.24	Fair
It is too far	1087	0.43	Moderate	924	0.37	Fair
The route is difficult to walk/run or bike because of garbage, water or bad smells	1092	0.35	Fair	915	0.24	Fair
The route is isolated	1083	0.50	Moderate	913	0.15	Slight
My child has a disability	1088	0.24	Slight	908	0.37	Fair

**Table 4:** Convergent validity by country and region (Urban) for the number of active trips to/from different destinations - parent vs. child report

	Urban (Kenya)		Urban (Nigeria)	
	N	Pearson Correlation (r)	N	Pearson Correlation (r)
School (Parent) vs School (Child)	376	.772^a^	252	.594^a^
Friends (Parent) vs Friends (Child)	375	.452^a^	239	.783^a^
Relatives (Parent) vs Relatives (Child)	370	.441^a^	237	.887^a^
Park (Parent) vs Park (Child)	373	.488^a^	237	.779^a^
Shop (Parent) vs Shop (Child)	376	.593^a^	243	.691^a^
Sport (Parent) vs Sport (Child)	367	.445^a^	234	.803^a^
Faith (Parent) vs Faith (Child)	374	.460	247	.810^a^
AT from Destinations				
School (Parent) vs School (Child)	371	.706^a^	250	.615^a^
Friends (Parent) vs Friends (Child)	370	.503^a^	238	.797^a^
Relatives (Parent) vs Relatives (Child)	368	.453^a^	236	.893^a^
Park (Parent) vs Park (Child)	370	.482^a^	236	.780^a^
Shop (Parent) vs Shop (Child)	373	.619^a^	241	.712^a^
Sport (Parent) vs Sport (Child)	362	.488^a^	233	.825^a^
Faith (Parent) vs Faith (Child)	369	.510^a^	245	.831^a^

^a^Correlation is significant at the 0.01 level (2-tailed)

**Table 5:** Convergent validity by country and region (peri-urban) for the number of active trips to/from different destinations - parent vs. child report

	Peri-urban (Kenya)		Peri-urban (Nigeria)	
	N	Pearson Correlation (r)	N	Pearson Correlation (r)
School (Parent) vs School (Child)	369	.064	349	.744^a^
Friends (Parent) vs Friends (Child)	368	.095	329	.813^a^
Relatives (Parent) vs Relatives (Child)	359	.093	328	.844^a^
Park (Parent) vs Park (Child)	365	−.045	330	.825^a^
Shop (Parent) vs Shop (Child)	368	.049	331	.808^a^
Sport (Parent) vs Sport (Child)	360	.047	322	.814^a^
Faith (Parent) vs Faith (Child)	367	−.029	339	.798^a^
AT from Destinations				
School (Parent) vs School (Child)	369	−.058	348	.708^a^
Friends (Parent) vs Friends (Child)	368	.036	327	.812^a^
Relatives (Parent) vs Relatives (Child)	360	−.018	328	.829^a^
Park (Parent) vs Park (Child)	365	−.036	330	.828^a^
Shop (Parent) vs Shop (Child)	368	−.015	330	.812^a^
Sport (Parent) vs Sport (Child)	360	.003	322	.800^a^
Faith (Parent) vs Faith (Child)	367	−.084	340	.845^a^

^a^Correlation is significant at the 0.01 level (2-tailed)

**Table 6:** Convergent validity by country and region (Rural) for the number of active trips to/from different destinations - parent vs. child report

	Rural (Kenya)		Rural (Nigeria)	
	N	Pearson Correlation (r)	N	Pearson Correlation (r)
School (Parent) vs School (Child)	353	.999	287	.317^a^
Friends (Parent) vs Friends (Child)	353	1.000	265	.599^a^
Relatives (Parent) vs Relatives (Child)	353	1.000	276	.452^a^
Park (Parent) vs Park (Child)	353	1.000	260	.664^a^
Shop (Parent) vs Shop (Child)	353	1.000	274	.635^a^
Sport (Parent) vs Sport (Child)	353	1.000	266	.494^a^
Faith (Parent) vs Faith (Child)	353	1.000	275	.682^a^
AT from Destinations				
School (Parent) vs School (Child)	353	1.000	287	.288^a^
Friends (Parent) vs Friends (Child)	353	1.000	266	.592^a^
Relatives (Parent) vs Relatives (Child)	353	1.000	275	.443^a^
Park (Parent) vs Park (Child)	353	1.000	259	.674^a^
Shop (Parent) vs Shop (Child)	353	1.000	276	.633^a^
Sport (Parent) vs Sport (Child)	353	1.000	267	.487^a^
Faith (Parent) vs Faith (Child)	353	1.000	274	.707^a^

^a^Correlation is significant at the 0.01 level (2-tailed)

**Table 7:** Combined convergent validity for the two countries (Kenya and Nigeria) by region for the number of active trips to/from different destinations - parent vs. child report

	Urban		Peri-urban		Rural	
	N	Pearson Correlation	N	Pearson Correlation	N	Pearson Correlation
School (Parent) vs School (Child)	628	.678^a^	718	.320^a^	640	.654^a^
Friends (Parent) vs Friends (Child)	614	.559^a^	697	.545^a^	618	.833^a^
Relatives (Parent) vs Relatives (Child)	607	.644^a^	687	.510^a^	629	.814^a^
Park (Parent) vs Park (Child)	610	.577^a^	695	.459^a^	613	.882^a^
Shop (Parent) vs Shop (Child)	619	.615^a^	699	.383^a^	627	.847^a^
Sport (Parent) vs Sport (Child)	601	.526^a^	682	.483^a^	619	760^a^
Faith (Parent) vs Faith (Child)	621	.795^a^	706	.500^a^	628	.767^a^
AT from Destinations						
School (Parent) vs School (Child)	621	.674^a^	717	.240^a^	640	.646^a^
Friends (Parent) vs Friends (Child)	608	.590^a^	695	.440^a^	619	.832^a^
Relatives (Parent) vs Relatives (Child)	604	.625^a^	688	.407^a^	628	.805^a^
Park (Parent) vs Park (Child)	606	.569^a^	695	.488^a^	612	.883^a^
Shop (Parent) vs Shop (Child)	614	.643^a^	698	.395^a^	629	.845^a^
Sport (Parent) vs Sport (Child)	595	.560^a^	682	.410^a^	620	.762^a^
Faith (Parent) vs Faith (Child)	614	.820^a^	707	.533^a^	627	.787^a^

^a^Correlation is significant at the 0.01 level (2-tailed)

## Discussion

This study described the development and convergent validity of new child and parent AT questionnaires for children from three African countries. Through a rigorous procedure that involved an environmental scan of existing AT tools, a systematic review of African and non-African studies and consensus discussions within the research team, we developed child and parent AT questionnaires that demonstrate moderate to substantial convergent validity, at least for measuring the number of active trips to/from a range of destinations, and these questionnaires could be used for research purposes in Africa. Because transportation infrastructure, travel patterns and physical activity behaviours of African children are different from those in the developed countries [20], it is important to tailor or adapt AT measures to the African context before they could be used to assess the travel behaviours of children in Africa.

Our AT questionnaires demonstrated acceptable convergent validity for questions on the number of trips to and from various destinations among Kenyan and Nigerian children. The correlations between the main travel mode to and from school across seasons were very high suggesting that there may be no substantial seasonal variations in the mode of travel to school in African children from these three countries. This is not surprising given that seasonal variations in weather are not normally as dramatic as is the case in countries in North America and Europe where one may expect more seasonal variations. Furthermore, African children may need to walk long distances to and from school because they have limited access to alternative modes of transportation, especially in rural areas. For example, in their study conducted in Ghana, Malawi, and South Africa, Porter et al. [21] reported that a large proportion of children/youth engaged in AT despite their concerns about safety. Nevertheless, motorized travel (e.g., cars, buses and motorcycles) is common in urban areas of Africa. While AT is a dominant mode of travel to and from school among rural youth in Africa, passive transportation (cars, buses and motorcycles) is a common mode of travel to and from school among their urban counterparts [6, 22–24].

Overall, the convergent validity was fair to moderate for most items on the section on perceived barriers to AT. These results could reflect different perceptions of barriers to AT between parents and children and/or lack of awareness on the part of parents regarding their child’s route to school. One would expect that differences in perceptions and awareness would result in lower agreement between child and parent responses and, hence, lower kappa coefficients. Qualitative exploration of what constitutes environmental barriers to AT among children in Africa and how they vary between children and parents could be an important future research direction, especially given the high burden of road injuries in Africa [25]. The low convergent validity observed between child and parent reports for the questions on perceived barriers to AT in the pilot-study may also be due to the limited variability of children and parent ratings. Barriers to active transportation are known to be more challenging to measure than school travel mode [26].

It is important to note the large between country variations in findings when interpreting the results. Our data suggest that convergent validity was comparatively better in Nigeria than Kenya for most items on AT to/from various destinations, but better in Kenya than Nigeria for most items on challenges/barriers to AT to school. Perhaps, there are subtle behavioural or social norms that influence perceptions of destinations and barriers to AT of children between countries in Africa. Plausibly, agreement between parents and children was stronger for destinations like faith places, sport venues (soccer fields) and shops or markets in Nigeria than Kenya, perhaps because the social responsibility for parents to accompany children to these ubiquitous destinations is stronger in Nigeria. Similarly, the poor convergent validity in Nigeria for challenges/barriers items that focused on bicycle safety and boring route could suggest that these barriers are not as contextually dominant for Nigeria compared to Kenya. Nevertheless, the between country differences in our findings highlight the need for country specific attention to item refinement when applying the instruments across Africa.

Although we found good evidence of convergent validity by regions (urban, peri-urban and rural) for most items on AT to/from various destinations in the pooled analysis, validity coefficients appear to be stronger in the urban and rural regions than the peri-urban region. This finding reflects the possibility that the interpretation of items on our questionnaire between parents and children may be different across levels of urbanization in Africa. Moreover, the country specific analysis by region revealed that while convergent validity was good across regions in Nigeria, it was only good in urban region in Kenya. Absence of evidence of convergent validity for the questionnaire in peri-urban and rural regions of Kenya could be due to the fact that children in peri-urban regions of Kenya have more complex travel patterns due to the need to access destinations that are further away. Most children therefore would use mixed modes of transport including walking, running and/or motorized means to their destination.

### Strengths and limitations of the study

To our knowledge this is the first study to develop and validate questionnaires for assessing child AT to and from a broad range of destinations among African children. The development of questionnaires to measure child AT in Africa is important, especially in light of the current physical activity transition that is taking place in the continent [25]. Assessing convergent validity by examining responses from both the children and their parents/guardians is also a strength of this study. Other studies have often included only parental reports (see Larouche et al. [9] for review). We examined travel behaviour to and from a much wider range of destinations compared to previous studies, which predominantly focussed on the trip to/from school. Finally, the large sample recruited from three African countries for the main study was another strength.

Limitations of the study included i) the use of a convenience sample that limits the generalizability of our study; ii) failure to ask participants to write “0” if they did not travel to a particular destination (which resulted in a very large amount of missing data); iii) Some missing data in Kenya an Nigeria which may reflect response bias; iv) large missing data for Mozambique in the main study which may limit generalizability of findings to children in that country; v) low convergent validity for many of the items on perceived barriers to AT, suggesting that future qualitative research may be needed to better understand which variables may hinder AT in the African context; and vi) we were not able to examine the test-retest reliability of our questionnaires, so future studies will be needed to address this limitation.

## Conclusions

The new self-administered AT questionnaires were found to have acceptable convergent validity between children’s reports to parents’ reports for assessing AT behaviours in a large, diverse sample of African children from three different countries representing various African regions. We found important between country differences in convergent validity indicating the need for country-specific attention when applying the questionnaires. The questionnaires may be appropriate for use in other African countries to assess AT among African school-aged children, but there remains a clear need for further research to improve the measurement of barriers to AT. Future studies should examine the test-retest reliability of these new questionnaires.

## Additional files

Additional file 1: Active transport and physical activity assessment tool for African school children. (DOCX 26 kb)

Additional file 2: **Table S1.** Correlations between the main travel mode to home by season - parent report (pilot study, *n* = 96). **Table S2.** Correlations between the main travel mode to school by season - parent report (pilot study, *n* = 96). **Table S3.** Correlations between the main travel mode to home by season - child report (pilot study, *n* = 115). **Table S4.** Correlations between the main travel mode to school by season - child report (pilot study, *n* = 115). (DOCX 17 kb)

## Abbreviations

ALO: Adewale Luqman Oyeyemi
AP: Antonio Prista
AT: Active transportation
GEO: George Evans Owino
ICC: Intraclass correlation coefficient
IDRC: International Development Research Centre
KKA: Kingsley Kolapo Akinroye
MST: Mark Stephen Tremblay
MVPA: Moderate-to-vigorous physical activity
NY: New York
PABAK: Prevalence-adjusted bias-adjusted kappa
RL: Richard Larouche
SH: Sylvester Heyker
SPSS: Statistical Package for the Social Science
USA: United States of America
VOO: Vincent Ochieng Onywera

## References

[1] World Health Organization (WHO) (2010). Global recommendations on physical activity for health.

[2] Colley RC, Garriguet D, Janssen I, Craig CL, Clarke J, Tremblay MS (2011). Physical activity of Canadian children and youth: accelerometer results from the 2007 to 2009 Canadian health measures survey. Health Reps.

[3] Troiano RP, Berrigan D, Dodd KW, Mâsse LC, Tilert T, McDowell M (2008). Physical activity in the United States measured by accelerometer. Med Sci Sports Exerc.

[4] Guthold R, Cowan MJ, Autenrieth CS, Kann L, Riley LM (2010). Physical activity and sedentary behavior among school-children: a 34-country comparison. J Pediatr.

[5] Hallal P, Andersen LB, Bull FC, Guthold R, Haskell W, Ekelund U (2012). For the lancet physical activity series working group. Global physical activity levels: surveillance progress, pitfalls and prospects. Lancet.

[6] Tremblay MS, Gray CE, Akinroye KK, Harrington DM, Katzmarzyk PT, Estelle V (2014). Physical activity of children: a global matrix of grades comparing 15 countries. J Phys Act Health.

[7] Pahkala K, Heinonen OJ, Simell O, Viikari JS, Rönnemaa T, Niinikoski H (2011). Association of physical activity with vascular endothelial function and intima-media thickness. Circulation.

[8] Lee IM, Shiroma EJ, Lobelo F, Puska P, Blair SN, Katzmarzyk PT (2012). Effect of physical inactivity on major non-communicable diseases worldwide: an analysis of burden of disease and life expectancy. Lancet.

[9] Larouche R, Oyeyemi AL, Prista A, Onywera VO, Akinroye KK, Tremblay MS (2014). A systematic review of active transportation research in Africa and the psychometric properties of measurement tools in children and youth. Int J BehavNutr Phys Act.

[10] Forman H, Kerr J, Norman GJ, Saelens BE, Durant NH, Harris SK (2008). Reliability and validity of destination-specific barriers to walking and cycling for youth. Prev Med.

[11] Larouche R, Barnes J, Tremblay MS (2013). Too far to walk or bike?. Can J Public Health.

[12] Larouche R, Faulkner G, Fortier M, Tremblay MS (2014). Active transportation and adolescents’ health: the Canadian health measures survey. Am J Prev Med.

[13] Brook RD, Rajagopalan S, Pope CA, Brook JR, Bhatnagar A, Diez-Roux AV (2010). Particulate matter air pollution and cardiovascular disease: an update to the scientific statement from the American Heart Association. Circulation.

[14] Oyeyemi AL, Ishaku CM, Oyekola J, Wakawa HD, Lawan A, Yakubu S (2016). Patterns and associated factors of physical activity among adolescents in Nigeria. PLoS One.

[15] Cohen J (1960). A coefficient of agreement for nominal scales. Educ Psychol Meas.

[16] Cohen J (1968). Weighted kappa: nominal scale agreement provision for scaled disagreement or partial credit. Psychol Bull.

[17] Hallgren KA (2012). Computing inter-rater reliability for observational data: an overview and tutorial. Tutor Quant Methods Psychol.

[18] Landis JR, Koch GG (1977). The measurement of observer agreement for categorical data. Biometrics.

[19] Feinstein AR, Cicchetti DV (1990). High agreement but low kappa: I. The problems of two paradoxes. J Clin Epidemiol.

[20] Muthuri SK, Wachira LJ, Onywera VO, Tremblay MS (2016). Associations between parental perceptions of the neighborhood environment and childhood physical activity: results from ISCOLE-Kenya. J Phys Act Health.

[21] Porter G, Hampshire K, Abane A, Robson E, Munthali A, Mashiri M, Tanle A (2010). Moving young lives: mobility, immobility and inter-generational tensions in urban Africa. Geoforum.

[22] Tremblay MS, Barnes JD, González SA, Katzmarzyk PT, Onywera VO, Reilly JJ (2016). Global matrix 2.0: report card grades on the physical activity of children and youth comparing 38 countries. J Phys Act Health.

[23] Onywera VO, Adamo KB, Sheel AW, Waudo JN, Boit MK, Tremblay M (2012). Emerging evidence of the physical activity transition in Kenya. J Phys Act Health.

[24] Ojiambo RM, Easton C, Casajus JA, Konstabel K, Reilly JJ, Pitsiladis Y (2012). Effect of urbanization on objectively measures physical activity levels, sedentary time, and indices of adiposity in Kenyan adolescents. J Phys Act Health.

[25] Goenka S, Andersen LB (2016). Urban design and transport to promote healthy lives. Lancet.

[26] McDonald NC, Dwelley AE, Combs TS, Evenson KR, Winters RH (2011). Reliability and validity of the safe routes to school parent and student surveys. Int J Behav Nutr Phys Act.

