# Supplementary material for: Development and convergent validity of new self-administered questionnaires of active transportation in three African countries: Kenya, Mozambique and Nigeria
Source: BMC Public Health. 2018 Aug 16;18:1018. doi: 10.1186/s12889-018-5954-z (PMC6097429; doi:10.1186/s12889-018-5954-z)
Supplement: Supplementary file 1 — Active transport and physical activity assessment tool for African school children. (DOCX 26 kb) [file 12889_2018_5954_MOESM1_ESM.docx]

**Additional File 1: ACTIVE TRANSPORT AND PYSICAL ACTIVITY ASSEMENT TOOL FOR AFRICAN SCHOOL CHILDREN**

**Instructions:** The child questionnaire is to be completed in the classroom under the supervision of research assistants. The transportation diary is to be completed in the classroom for each school day during the week when they wear the pedometer. The parental questionnaire is to be completed by a parent (or guardian) of the child. Parents should only be asked to complete the transportation diary and the items on their child’s usual mode of transportation to various destinations.

**PARENTAL QUESTIONNAIRE**

**Country: ______________School: _____________Urban/Semi-urban/rural:_____________**

**Date:____________________________**

**Study ID #:_____________________________________**

1. What is your relationship to the child? (are you the Mother/Father/Guardian?)

____________________________

2. What is highest level of education achieved by any of the mother/father/guardian in this home?

- Less than primary school
- Primary school
- Less than high school
- Some high school
- High school
- Diploma/Higher Diploma
- Bachelor’s degree
- Graduate (Masters/PhD)/professional degree

3. How many functioning vehicles (cars or trucks) are available for use at your house?

- 0
- 1
- 2
- 3
- 4 or more

4. How many functioning motorcycles (piki-piki) [or tricycles] are available for use at your house?

- 0
- 1
- 2
- 3
- 4 or more

5. How many functioning bicycles are available for use at your house?

- 0
- 1
- 2
- 3
- 4 or more

6. How does your child usually go **to school** in a typical week (from Monday to Friday)? Please tick only one box.

□ He/she walks □ He/she bikes □ He/she runs

□By car or van □ By bus or train □ By motorcycle

□By another way. Please write it down: _______________________

7. How does your child usually go **back home** in a typical week (from Monday to Friday)? Please tick only one box.

□ He/she walks □ He/she bikes □ He/she runs

□By car or van □ By bus or train □ By motorcycle

□By another way. Please write it down: _______________________

8. How far away is your child’s school from your home?___________ kilometers

9. Are any of the following a challenge/barrier for your child to walk or run or bike to school?

| **Item** | **1**  **Yes** | **2**  **No** |
| --- | --- | --- |
| There are too many hills along the way |  |  |
| There are no suitable walking/running or biking paths |  |  |
| The route is boring (nothing interesting to see) |  |  |
| The route does not have good lighting |  |  |
| There is too much traffic along the route |  |  |
| There are dangerous crossings |  |  |
| My child gets too hot and sweaty |  |  |
| No other children walk/run or bike to school |  |  |
| It’s not considered fashionable to walk/run or bike |  |  |
| My child has too many things to carry |  |  |
| It is easier for me to drive my child |  |  |
| It involves too much planning ahead |  |  |
| It is unsafe because of crime (strangers, gangs, drugs) |  |  |
| My child gets bullied, teased, harassed |  |  |
| There is nowhere to leave a bike safely |  |  |
| There are stray dogs or other dangerous animals |  |  |
| It is too far |  |  |
| The route is difficult to walk/run or bike because of garbage, water or bad smells |  |  |
| The route is isolated |  |  |
| My child has a disability |  |  |
| Please indicate any other challenges/barriers: | | |

10. When your child wore the pedometer, how many times did he/she go to and from home to the following destinations using active modes of travel (for example, walking, running, biking)? Please respond for each day of the week.

| Destinations | Monday | | Tuesday | | Wednesday | | Thursday | | Friday | | Saturday | | Sunday | |
| --- | --- | --- | --- | --- | --- | --- | --- | --- | --- | --- | --- | --- | --- | --- |
|  | To | From | To | From | To | From | To | From | To | From | To | From | To | From |
| School |  |  |  |  |  |  |  |  |  |  |  |  |  |  |
| Friend’s houses/home |  |  |  |  |  |  |  |  |  |  |  |  |  |  |
| Relative’s houses/home |  |  |  |  |  |  |  |  |  |  |  |  |  |  |
| Parks or playgrounds |  |  |  |  |  |  |  |  |  |  |  |  |  |  |
| Shops or markets, or restaurants |  |  |  |  |  |  |  |  |  |  |  |  |  |  |
| Sport venues (e.g soccer field, swimming pool) |  |  |  |  |  |  |  |  |  |  |  |  |  |  |
| Faith places (e.g church, mosque) |  |  |  |  |  |  |  |  |  |  |  |  |  |  |
| Other** | | | | | | | | | | | | | | |

** If you have written “other”, please specify which destination it is: ____________________

**CHILD QUESTIONAIRE**

Country: ______________School: ___________________ Urban/Semi-urban/rural:_____________

**Date:____________________________**

**Study ID #:______________________________________**

Age:_________________________________________________________________________

­­­­Gender (boy/girl): ______________________________________________________________

1. How do you usually go to school in a typical week (from Monday to Friday)? Please tick only one box.

□ I walk □ I bike □ I run

□By car or van □ By bus or train □ By motorcycle

□By another way. Please write it down: _______________________

2. How do you usually go back home in a typical week (from Monday to Friday)? Please tick only one box.

□ I walk □ I bike □ I run

□By car or van □ By bus or train □ By motorcycle

□By another way. Please write it down: _______________________

3. How far away is your school from your home? ___________ kilometers

4. Are any of the following a challenge/barrier for you to walk or run or bike to school?

| **Item** | **1**  **Yes** | **2**  **No** |
| --- | --- | --- |
| There are too many hills along the way |  |  |
| There are no suitable walking/running or biking paths |  |  |
| The route is boring (nothing interesting to see) |  |  |
| The route does not have good lighting |  |  |
| There is too much traffic along the route |  |  |
| There are dangerous crossings |  |  |
| I get too hot and sweaty |  |  |
| No other children walk/run or bike to school |  |  |
| It’s not considered fashionable to walk/run or bike |  |  |
| I have too many things to carry |  |  |
| It is easier for my parents to drive me |  |  |
| It involves too much planning ahead |  |  |
| It is unsafe because of crime (strangers, gangs, drugs) |  |  |
| I get bullied, teased, harassed |  |  |
| There is nowhere to leave a bike safely |  |  |
| There are stray dogs or other dangerous animals |  |  |
| It is too far |  |  |
| The route is difficult to walk/run because of garbage, water or bad smells |  |  |
| The route is isolated |  |  |
| I have a disability |  |  |
| Please indicate any other challenges/barriers: | | |

**CHILD TRANSPORT DIARY**

**Country: ______________School: ___________________ Urban/Semi-urban/rural:_____________**

**Date:_____________________________**1. Study ID #:___________________________________­­­­2. Gender (boy/girl): ______________________

5. When you wore the pedometer, how many times did you go to and from **home to the following destinations** using active modes of travel such as walking, running, biking)? Please respond for each day of the week.

| Destinations | Monday | | Tuesday | | Wednesday | | Thursday | | Friday | | Saturday | | Sunday | |
| --- | --- | --- | --- | --- | --- | --- | --- | --- | --- | --- | --- | --- | --- | --- |
|  | To | From | To | From | To | From | To | From | To | From | To | From | To | From |
| School |  |  |  |  |  |  |  |  |  |  |  |  |  |  |
| Friend’s houses/home |  |  |  |  |  |  |  |  |  |  |  |  |  |  |
| Relative’s houses/home |  |  |  |  |  |  |  |  |  |  |  |  |  |  |
| Parks or playgrounds |  |  |  |  |  |  |  |  |  |  |  |  |  |  |
| Shops or markets, or restaurants |  |  |  |  |  |  |  |  |  |  |  |  |  |  |
| Sport venues (e.g soccer field, swimming pool) |  |  |  |  |  |  |  |  |  |  |  |  |  |  |
| Faith places (e.g church, mosque) |  |  |  |  |  |  |  |  |  |  |  |  |  |  |
| Other** | | | | | | | | | | | | | | |

** If you have written “other”, please specify which destination it is: ____________________

6. For each day of the week, please write “yes” if you have worn the activity monitor for most of the day or “no” if you did not.

|  | Monday | Tuesday | Wednesday | Thursday | Friday | Saturday | Sunday |
| --- | --- | --- | --- | --- | --- | --- | --- |
| Did you wear the pedometer for most of the day? |  |  |  |  |  |  |  |
