# Supplementary material for: Development and convergent validity of new self-administered questionnaires of active transportation in three African countries: Kenya, Mozambique and Nigeria
Source: BMC Public Health. 2018 Aug 16;18:1018. doi: 10.1186/s12889-018-5954-z (PMC6097429; doi:10.1186/s12889-018-5954-z)
Supplement: Supplementary file 2 — Table S1. Correlations between the main travel mode to home by season - parent report (pilot study, n = 96). Table S2. Correlations between the main travel mode to school by season - parent report (pilot study, n = 96). Table S3. Correlations between the main travel mode to home by season - child report (pilot study, n = 115). Table S4. Correlations between the main travel mode to school by season - child report (pilot study, n = 115). (DOCX 17 kb) [file 12889_2018_5954_MOESM2_ESM.docx]

**Table S1.** Correlations between the main travel mode to home by season - parent report (pilot study, n=96)

|  | | | Home travel mode Jan-Apr | Home travel mode May-Aug | Home travel mode Sep-Dec |
| --- | --- | --- | --- | --- | --- |
| Spearman's rho | Home travel mode Jan-Apr | R | 1.000 | .956^**^ | .950^**^ |
|  | Home travel mode May-Aug | Correlation Coefficient | .956^**^ | 1.000 | .907^**^ |
|  |  | Sig. (2-tailed) | .000 |  | .000 |
|  | Home travel mode Sep-Dec | Correlation Coefficient | .950^**^ | .907^**^ | 1.000 |
|  |  | Sig. (2-tailed) | .000 | .000 |  |

**. Correlation is significant at the 0.01 level

**Table S2.** Correlations between the main travel mode to school by season - parent report (pilot study, n=96)

|  | | | Mode of travel Jan-Apr | Mode of travel May-Aug | Mode of travel mode Sep-Dec |
| --- | --- | --- | --- | --- | --- |
| Spearman's rho | Home travel mode Jan-Apr | Correlation Coefficient | 1.000 | .874^**^ | .827^**^ |
|  |  | Sig. (2-tailed) |  | .000 | .000 |
|  | Home travel mode May-Aug | Correlation Coefficient | .874^**^ | 1.000 | .871^**^ |
|  |  | Sig. (2-tailed) | .000 |  | .000 |
|  | Home travel mode Sep-Dec | Correlation Coefficient | .827^**^ | .871^**^ | 1.000 |
|  |  | Sig. (2-tailed) | .000 | .000 |  |

**. Correlation is significant at the 0.01 level

**Table S3.** Correlations between the main travel mode to home by season - child report (pilot study, n=115)

|  | | | Home travel mode Jan-Apr | Home travel mode May-Aug | Home travel mode Sep-Dec |
| --- | --- | --- | --- | --- | --- |
| Spearman's rho | Home travel mode Jan-Apr | Correlation Coefficient | 1.000 | .874^**^ | .827^**^ |
|  |  | Sig. (2-tailed) |  | .000 | .000 |
|  | Home travel mode May-Aug | Correlation Coefficient | .874^**^ | 1.000 | .871^**^ |
|  |  | Sig. (2-tailed) | .000 |  | .000 |
|  | Home travel mode Sep-Dec | Correlation Coefficient | .827^**^ | .871^**^ | 1.000 |
|  |  | Sig. (2-tailed) | .000 | .000 |  |

**. Correlation is significant at the 0.01 level

**Table S4.** Correlations between the main travel mode to school by season - child report (pilot study, n=115)

|  | | | Home travel mode Jan-Apr | Home travel mode May-Aug | Home travel mode Sep-Dec |
| --- | --- | --- | --- | --- | --- |
| Spearman's rho | Home travel mode Jan-Apr | Correlation Coefficient | 1.000 | .965^**^ | .888^**^ |
|  |  | Sig. (2-tailed) |  | .000 | .000 |
|  | Home travel mode May-Aug | Correlation Coefficient | .965^**^ | 1.000 | .879^**^ |
|  |  | Sig. (2-tailed) | .000 |  | .000 |
|  | Home travel mode Sep-Dec | Correlation Coefficient | .888^**^ | .879^**^ | 1.000 |
|  |  | Sig. (2-tailed) | .000 | .000 |  |

**. Correlation is significant at the 0.01 level
